# Thyroid antibody status, thyroid function, and the risk of coronary heart disease and stroke: an individual participant data analysis from 14 cohorts

**DOI:** 10.1093/ejendo/lvaf209

**Published:** 2025-10-30

**Authors:** Ola Hysaj, Orestis Efthimiou, Tinh-Hai Collet, Anne R. Cappola, Heba Alwan, Jacobijn Gussekloo, Layal Chaker, Maryam Kavousi, Fereidoun Azizi, Anna Köttgen, Elizabeth Selvin, Stella Trompet, John P. Walsh, Suzanne J. Brown, Massimo Iacoviello, Robin P.F. Dullaart, Stephan J.L. Bakker, José A. Sgarbi, Till Ittermann, Henry Völzke, Isabela M. Benseñor, Sergio Valdés, Cristina Maldonado-Araque, Bruce M. Psaty, Frank J. Wolters, Bjørn O. Åsvold, Cinzia Del Giovane, Nicolas Rodondi

**Affiliations:** 1 Institute of Primary Health Care (BIHAM), University of Bern, 3012 Bern, Switzerland; 2 Graduate School for Health Sciences, University of Bern, 3012 Bern, Switzerland; 3 Service of Endocrinology and Diabetology, Department of Medicine, Geneva University Hospitals (HUG), 1205 Geneva, Switzerland; 4 Diabetes Centre, Faculty of Medicine, University of Geneva, 1206, Switzerland; 5 Perelman School of Medicine, University of Pennsylvania, Philadelphia, PA 19104, United States; 6 Department of Public Health and Primary Care, Leiden University Medical Center, 2333 ZA Leiden, the Netherlands; 7 LUMC Center for Medicine for Older People, Leiden University Medical Center, 2333 ZA Leiden, the Netherlands; 8 Internal Medicine, Erasmus Medical Center, 3015 GD Rotterdam, the Netherlands; 9 Endocrine Research Center, Research Institute for Endocrine Sciences, Shahid Beheshti University of Medical Sciences, 1985717413 Tehran, Iran; 10 Department of Epidemiology, Johns Hopkins Bloomberg School of Public Health, Baltimore, MD 21205, United States; 11 Faculty of Medicine and Medical Center, Institute of Genetic Epidemiology, University of Freiburg, 79106 Freiburg, Germany; 12 Section Gerontology and Geriatrics, Department of Internal Medicine, Leiden University Medical Center, 2333 ZA Leiden, the Netherlands; 13 Discipline of Internal Medicine, University of Western Australia Medical School, Perth, WA 6009, Australia; 14 Department of Endocrinology and Diabetes, Sir Charles Gairdner Hospital, Perth, WA 6009, Australia; 15 Department of Medical and Surgical Sciences, University of Foggia, 71122 Foggia, Italy; 16 Department of Internal Medicine, University Medical Center Groningen, University of Groningen, 9700 RB Groningen, the Netherlands; 17 Faculdade de Medicina de Marília, 17519-030 Marilia, São Paulo, Brazil; 18 Institute for Community Medicine, Clinical-Epidemiological Research, University Medicine Greifswald, 17475 Greifswald, Germany; 19 Center of Clinical and Epidemiological Research, University Hospital of the University of São Paulo, 01246-903 São Paulo, Brazil; 20 Department of Endocrinology and Nutrition, Hospital Regional Universitario de Málaga/Universidad de Málaga, Instituto de Investigación Biomedica de Málaga (IBIMA), CIBERDEM, Instituto de Salud Carlos III, 29010 Malaga, Spain; 21 Cardiovascular Health Research Unit, Departments of Medicine, Epidemiology, and Health Systems and Population Health, University of Washington, Seattle, WA 98101, United States; 22 Department of Epidemiology, Erasmus Medical Center, 3015 GD Rotterdam, the Netherlands; 23 Department of Radiology, Erasmus Medical Center, 3015 GD Rotterdam, the Netherlands; 24 HUNT Center for Molecular and Clinical Epidemiology, Department of Public Health and Nursing, NTNU, Norwegian University of Science and Technology, 7491 Trondheim, Norway; 25 Department of Endocrinology, Clinic of Medicine, St. Olavs Hospital, Trondheim University Hospital, 7030 Trondheim, Norway; 26 Department of Medical and Surgical Sciences for Children & Adults, University of Modena, 41124 Modena, Italy; 27 Department of General Internal Medicine, Bern University Hospital (Inselspital), University of Bern, 3010 Bern, Switzerland

**Keywords:** thyroid peroxidase antibodies, cardiovascular disease, subclinical hypothyroidism, individual participant data analysis

## Abstract

**Objective:**

The association of thyroid peroxidase antibodies (TPOAb) status with cardiovascular disease (CVD) risk, independent of thyroid function, remains unclear. We aimed to determine whether positive TPOAb is associated with increased CVD risk, overall and among individuals with subclinical hypothyroidism.

**Methods:**

We conducted a 2-stage individual participant data analysis including cohort studies identified through the Thyroid Studies Collaboration and systematic database searches (MEDLINE, EMBASE, Cochrane Library) through November 2024. Primary outcomes were coronary heart disease (CHD) events, CHD mortality, stroke events, and stroke mortality. We used Cox proportional hazards models, adjusted for age, sex, and thyroid-stimulating hormone (TSH) within each cohort, followed by a random-effects meta-analysis to assess cardiovascular outcomes by TPOAb status, overall and in the subclinical hypothyroidism subgroup.

**Results:**

Among 100 250 adults from 14 cohort studies (median age 55 years, 56.7% women), 11.9% were TPOAb-positive, and 5.4% had subclinical hypothyroidism. In the overall population, we found no evidence of increased CVD risk in TPOAb-positive compared to TPOAb-negative individuals: Hazard ratio (HR) 1.00 (95% CI 0.90–1.11) for CHD events; HR 0.95 (95% CI 0.78–1.16) for CHD mortality; HR 0.98 (95% CI 0.87–1.11) for stroke events; and HR 1.06 (95% CI 0.81–1.40) for stroke mortality. In subclinical hypothyroidism, positive TPOAb was similarly not associated with increased risk for any of the CVD outcomes.

**Conclusions:**

Positive TPOAb status was not associated with increased risk of CHD or stroke. These findings do not support the use of TPOAb testing for cardiovascular risk assessment in the overall population or among individuals with subclinical hypothyroidism.

## Introduction

A positive test for thyroid peroxidase antibody (TPOAb), the most sensitive marker of thyroid autoimmunity, is present in 35%−65% of individuals with subclinical hypothyroidism and predicts progression to overt hypothyroidism.^1–5^ Both subclinical and overt hypothyroidism are associated with increased risk of cardiovascular disease (CVD), manifesting as functional cardiovascular abnormalities and atherosclerosis.^6–10^

Thyroid autoimmunity and atherosclerosis share key biological pathways, including chronic inflammation, oxidative stress, metabolic dysregulation, and endothelial dysfunction, that support the biological plausibility of an association between TPOAb and CVD.^10–13^ In addition, positive TPOAb status is not limited to individuals with thyroid dysfunction, and is detected in 10%−26% of euthyroid individuals.^14^ Associations between TPOAb and increased atherosclerotic changes, cardiometabolic risk factors, and carotid intima–media thickness have been reported in euthyroid individuals, suggesting that TPOAb itself, even in the absence of thyroid dysfunction, may contribute to cardiovascular risk.^15–18^

Despite these findings, few longitudinal studies have examined the relationship between TPOAb status and cardiovascular outcomes in subclinical hypothyroidism, and results are conflicting.^19–24^ While some studies have suggested that there is an increased risk, others suggest that this risk may be more closely related to the degree of thyroid dysfunction rather than the presence of TPOAb alone.^2,20,23,25^ These studies were underpowered with a low number of events, did not present subgroup analyses (eg, by TSH levels or age), and, most importantly, did not adequately account for confounding factors like TSH and other cardiovascular risk factors. Moreover, the role of TPOAb as an independent risk factor for cardiovascular events, irrespective of thyroid status, remains poorly understood. Only 1 population-based study has to date examined this question and was limited, however, due to the low number of CVD events.^25^

Understanding this relationship has clinical implications for the utility of TPOAb testing in cardiovascular risk assessment and the management of thyroid autoimmunity. This is particularly relevant for individuals with subclinical hypothyroidism, as physicians commonly factor TPOAb status when deciding whether to treat subclinical hypothyroidism, although guidelines differ in their recommendations regarding thyroid TPOAb measurement to better identify candidates for levothyroxine therapy.^26^

To address this gap, we conducted an individual participant data (IPD) meta-analysis of 14 prospective cohort studies to determine whether a positive TPOAb test is independently associated with cardiovascular events, such as coronary heart disease (CHD) and stroke. We further assessed if positive TPOAb status modifies risk for CHD and stroke among individuals with subclinical hypothyroidism.

## Methods

### Data sources and study selection

This IPD analysis was registered with the International Prospective Register of Systematic Reviews (PROSPERO; registration number CRD42023368755) and is reported in accordance with the Preferred Reporting Items for Systematic Reviews and Meta-Analyses of Individual Participant Data (PRISMA-IPD) guidelines.^27^ We initially identified studies through the Thyroid Studies Collaboration (TSC), a consortium of 29 prospective cohort studies with data on thyroid function and related outcomes.^8^ To be eligible for inclusion, studies were required to have baseline measurements of thyroid status (TSH, FT4) and TPOAb and cardiovascular outcomes (incident or fatal CHD and/or stroke). Nine cohorts from the TSC met the inclusion criteria. Previous publications provide a more detailed description of the included study designs.^2,6,24,25,28–33^ We additionally conducted a systematic literature review of the Embase, Cochrane Library, and MEDLINE (Ovid) databases with no language restriction, from inception to November 2024, to identify new cohorts; details of the search strategy are available in the supplement. Four out of the 9 cohorts that the literature review identified consented to collaborating and sharing data.^34–37^ We also included publicly available data from the National Health and Nutrition Examination Survey waves of 1999–2002 and 2011–2012.^38^ We excluded studies that solely included individuals taking thyroid-altering medication (antithyroid medications, thyroxine replacement, or amiodarone) or with overt hypothyroidism, defined as low FT4 and elevated TSH concentrations. Two reviewers (O.H. and H.A.) independently assessed eligibility for study inclusion, and a third reviewer (C.D.G.) resolved any discrepancies.

This research complied with the ethical standards of the Declaration of Helsinki. Institutional review boards approved all studies and secured written informed consent from all participants in their respective original studies. The Research Ethics Committee of the University of Bern waived the need for ethical approval given the retrospective nature of the study, which only used previously collected data and did not involve interventions.

### Data extraction and quality assessment

All the eligible studies provided individual-level data on thyroid function at baseline (TSH, FT4), TPOAb, demographics (age, sex), history of cardiovascular disease and risk factors (smoking, diabetes mellitus, systolic blood pressure, total cholesterol, and body mass index (BMI)), medication use (thyroid-altering medication, antihypertensive medication, and lipid-lowering medication), and outcomes (CHD events, CHD mortality, stroke events, and stroke mortality).

We assessed study quality and risk of bias using the Newcastle–Ottawa Scale after considering additional information received from study authors, such as methods of outcome adjudication and ascertainment, accounting for confounders, and completeness of follow-up ascertainment.^39^ Two reviewers, O.H. and H.A., rated all studies for quality.

### Thyroid function

Consistent with previous TSC studies, we classified TSH levels between 0.45 and 4.49 mIU/L as within the euthyroid range.^8,40–42^ Subclinical hypothyroidism was defined as a TSH between 4.50 and 19.99 mIU/L with FT4 within the reference range or elevated TSH without FT4 measurements due to the low likelihood of overt hypothyroidism in such cases.^14^ One of the eligible cohorts, Study of Health in Pomerania (SHIP), initiated an iodine fortification program a few years prior to participant inclusion; consequently, we applied a study-specific TSH reference range of 0.25–2.12 mIU/L, as recommended for iodine-deficient regions.^29,43^ Additionally, we conducted a sensitivity analysis that excluded this study.

We further stratified subclinical hypothyroidism into categories by TSH level: 4.5–6.9 mIU/L, 7.0–9.9 mIU/L, and 10.0–19.9 mIU/L. For FT4 and TPOAb status (positive vs. negative), we used cohort and assay-specific cutoffs, accounting for inter-study variability in measurement methods (further details in the Supplementary material). In a sensitivity analysis, we also excluded people with missing FT4 values.

### Definition of outcomes

In line with our prior research, we aimed to limit heterogeneity in the definition of the outcomes observed in some meta-analyses in the field by using more homogeneous outcome definitions.^8,20^ A CHD event was defined as a nonfatal myocardial infarction, coronary artery revascularization, incident angina pectoris, or hospital diagnosis of CHD or CHD-related mortality.^44^ CHD mortality was defined as deaths specifically attributed to CHD or sudden cardiac death.^8^ Stroke events combined fatal and nonfatal ischemic or hemorrhagic strokes based on the World Health Organization (WHO) definition as a clinical syndrome characterized by rapidly developing signs of focal (or, at times, global) disturbance of cerebral function lasting more than 24 hours or leading to death with no apparent cause other than that of vascular origin. The Supplementary material provides detailed definitions of CHD, stroke, events, and mortality for each cohort.

### Statistical analysis

We used a 2-step meta-analysis approach to investigate the association between TPOAb status (positive vs. negative) and each CVD outcome in the overall study population.^45^ We first fit separate Cox proportional hazard models to the data from each cohort. We then pooled study-specific estimates of HRs using a random-effects inverse-variance meta-analysis model.^46^ We opted for a random-effects model to incorporate expected heterogeneity of the association across settings in the estimation.^46^ Time to event was calculated for each outcome as time from baseline to first event. Data were censored at the time when a participant was lost to follow-up, died, or reached the end of the follow-up period without having an event. Participants were included in the analysis for all outcomes irrespective of previous nonfatal events unless stated otherwise. Results were summarized using forest plots (Review Manager version 5.0.24, Nordic Cochrane Centre, Copenhagen, Denmark). Between-study heterogeneity of log HRs was assessed by the *I*^2^ statistic.^47^

Primary analyses were adjusted for age, sex, and log-transformed TSH. We considered the age-, sex-, and TSH-adjusted model as the primary analysis because some traditional risk factors could be potential mediators of the relationship between TPOAb and cardiovascular disease rather than confounders.^48^ A fully adjusted model included additional adjustment for smoking status (never, former, or current smokers), BMI, systolic blood pressure, diabetes status, and total cholesterol. We selected these potential confounders based on our subject matter knowledge, prior literature in the field, and data availability.^49–51^ To address missing data, we applied a predefined threshold for imputation. Specifically, if a variable had more than 10% of data missing, we performed multiple imputations using chained equations under the assumption that data were missing at random. Imputations were conducted separately within each cohort, using the set of covariates available in that cohort, to ensure that imputed values reflected cohort-specific distributions. This approach allowed us to preserve statistical power and reduce potential bias that might arise from excluding participants with incomplete information. For variables with less than 10% missingness, we considered the impact on estimates to be negligible; therefore, we restricted analyses to participants with complete data for those variables. To explore sources of heterogeneity, we performed stratified analyses by age category (<65 and ≥65 years) and sex. We investigated effect modification using interaction tests for individual characteristics (age category, sex). Additionally, we conducted several sensitivity analyses to test the robustness of our results: (1) excluding thyroid-altering medication use at baseline; (2) excluding thyroid-altering medication use at baseline and follow-up; (3) excluding participants with a history of CVD; (4) excluding cohorts with recent iodine supplementation (SHIP); (5) further adjustment for lipid and hypertension medication; (6) only including studies that had formal adjudication procedures for CHD and stroke outcomes; (7) excluding studies with low quality based on quality assessment; (8) limiting analyses to euthyroid individuals.

In a secondary analysis, we assessed whether CVD risk differed by TPOAb status in individuals with subclinical hypothyroidism. Furthermore, to compare our results with previous publications, we decided to compare the risks of positive and negative TPOAb subgroups of individuals with sub-clinical hypothyroidism with euthyroid participants, overall and stratified by TSH levels (4.5–6.9, 7.0–9.9, and 10.0–19.9 mIU/L).

We assessed the proportional hazards assumption of the Cox model at the study level using graphical methods based on Schoenfeld residuals. We visually assessed funnel plots of age-, TSH-, and sex-adjusted estimates when 10 studies or more were included in the analysis and used the Egger test to assess the impact of small study effects and publication bias.^52^ We used Stata (version 16) for all analyses.

## Results

A total of 14 cohorts met the inclusion criteria and provided individual participant data for this analysis (Figure S1). The final sample included 100 250 adults (median age 55 years; 56.7% women), with a median follow-up of 12.1 years (inter-quartile range 6.6–14.7 years). At baseline, 11.9% (8155 participants) had positive TPOAb, and 5.4% (5412 participants) had subclinical hypothyroidism, of whom 41% (2221 participants) had positive TPOAb. Additionally, 2.9% (2715) of participants used thyroid medication at baseline, increasing to 6.1% (1695 participants) during follow-up. Table 1 and Table S1 display detailed cohort characteristics.

Outcome data were available for 99 287 participants for CHD mortality across all but 1 cohort, in 68 671 participants across 10 cohorts for CHD events, 65 343 participants across 10 cohorts for stroke mortality, and 38 485 participants across 8 cohorts for stroke events. Table 2 and Figures S2–S6 detail event counts, cohort-specific hazard ratios, and confidence intervals for each outcome.

### Association between TPOAb status and CVD in the entire population

Age-, sex-, and TSH-adjusted analyses showed no evidence of risk difference in CHD events (HR 1.00; 95% CI 0.90–1.11) or CHD mortality (HR 0.95; 95% CI 0.78–1.16) between individuals with positive and negative TPOAb. Similarly, stroke events (HR 0.98; 95% CI 0.87–1.11) and stroke mortality (HR 1.06; 95% CI 0.81–1.40) showed no evidence of increased risk in positive TPOAb. There was no evidence of an association between positive TPOAb and any of the outcomes among each individual study (Figures S2–S6). We observed minimal to no heterogeneity across studies: CHD events (*I*^2^ = 32%), CHD mortality (*I*^2^ = 31%), stroke events (*I*^2^ = 0%), and stroke mortality (*I*^2^ = 0%). Stratified analyses by sex and age group showed consistent results, with no evidence of interactions (all *P* > .1). HRs for all outcomes were similar after further adjusting for smoking, BMI, systolic blood pressure, and total cholesterol in participants with complete information and after excluding thyroid medication users at baseline or during follow-up (Table 2). Sensitivity analyses adjusting further for baseline medication use (lipid-lowering and antihypertensive medication) and excluding studies with low quality or recent iodine supplementation yielded comparable results. Similar findings were observed when the analysis was restricted to euthyroid individuals, with estimates showing no evidence of differences by TPOAb status (Tables S2 and S3).

### TPOAb status and CVD risk in subclinical hypothyroidism

Among participants with subclinical hypothyroidism, in age-, sex-, and TSH-adjusted analyses, we did not find evidence of differences in the CHD (events and mortality) and stroke mortality risks between those with positive and negative TPOAb compared to euthyroid individuals. For CHD events, HRs were 1.02 (95% CI 0.85–1.22) for individuals with positive TPOAb and 1.13 (95% CI 0.97–1.32) for those with negative TPOAb, compared to the euthyroid state. For CHD mortality, the HRs were 1.18 (95% CI 0.81–1.72) for TPOAb-positive individuals and 1.23 (95% CI 1.03–1.48) for TPOAb-negative individuals. For stroke mortality, the corresponding HRs were 1.14 (95% CI 0.67–1.93) for sub-clinical hypothyroidism with positive TPOAb and 1.33 (95% CI 0.87–2.04) for subclinical hypothyroidism with negative TPOAb compared to euthyroid individuals. For stroke events, HR was 0.94 (95% CI 0.70–1.23) for individuals with positive TPOAb compared to euthyroid. In contrast, subclinical hypothyroidism with negative TPOAb was associated with an elevated risk of stroke events compared to euthyroid individuals with an HR of 1.24 (95% CI 1.03–1.55).

Comparing individuals with subclinical hypothyroidism with positive versus negative TPOAb showed no evidence of risk differences for CHD events (HR 0.87; 95% CI 0.72–1.05), CHD mortality (HR 0.88; 95% CI 0.64–1.21), or stroke mortality (HR 0.93; 95% CI 0.51–1.90). However, positive TPOAb was associated with a lower risk of stroke events compared to negative TPOAb (HR 0.68; 95% CI 0.51–0.90) among individuals with subclinical hypothyroidism.

Risks for CHD events were consistent across age and sex subgroups (*P* for interaction > .14 for sex and *P* for interaction > .31 for age categories, Table 3), with no evidence of interactions by TPOAb status. Although risks for CHD mortality and stroke outcomes varied across age and sex subgroups, there was no evidence of interactions by TPOAb status (Table 4).

TSH-stratified analyses suggested increased risks for TSH levels of 10.0–19.9 mIU/L for CHD events among individuals with negative TPOAb (HR 1.67; 95% CI 1.03–2.69), CHD mortality among those with positive TPOAb (HR 1.80; 95% CI 1.13–2.87), and stroke mortality among those with negative TPOAb (HR 4.43; 95% CI 1.38–14.15), compared to euthyroid individuals. However, there was no evidence of differences by TPOAb status within each TSH category, and statistical evidence for trends was limited (*P* for interaction > .07 for all outcomes).

Sensitivity analyses that excluded participants using thyroid medications, adjusted for cardiovascular risk factors and medications in participants with complete cases, excluded participants with a history of CVD, or excluded studies with low quality or recent iodine supplementation were consistent with the primary analyses (Tables S3 and S4).

We did not find any evidence of publication bias for the TPOAb status and CVD analyses, either with a visual assessment of age-, sex-, and TSH-adjusted funnel plots or with the Egger test for all CHD events (*P* = .32), for CHD mortality (*P* = .41), for stroke events (*P* = .18), or for stroke mortality (*P* = .36). We did not find evidence against the proportional hazard assumption across studies (all *P* > .09), as shown in Table S4.

## Discussion

In this large IPD analysis of 14 prospective cohort studies, we found no evidence that a positive TPOAb status is independently associated in the overall study population with CVD outcomes, including CHD events, CHD mortality, stroke events, and stroke mortality, before or after adjustment for TSH and established cardiovascular risk factors. These results were consistent across various sensitivity, subgroup analyses, and exclusion criteria. Among individuals with subclinical hypothyroidism, positive TPOAb status was not associated with increased cardiovascular risk compared to euthyroid individuals. We did not find evidence of a difference in HR for CHD events, CHD, or stroke mortality outcomes. In contrast, in individuals with subclinical hypothyroidism, positive TPOAb status was linked to a lower risk of stroke events compared to negative TPOAb, and negative TPOAb was associated with an increased risk of stroke compared to euthyroid individuals.

To our knowledge, this is the first IPD analysis to assess the relationship between TPOAb status and cardiovascular outcomes, accounting for TSH and cardiovascular risk factors in a comprehensive manner in the overall population. Our findings are consistent with those of Khan et al.^25^, who similarly found no independent association between TPOAb status and cardiovascular mortality in their population-based study.

Consistent with some single-cohort studies, we found no association between positive TPOAb and CHD risk in individuals with subclinical hypothyroidism.^2,10,24^ However, the Rotterdam Study reported an association between positive TPOAb and myocardial infarction in women with subclinical hypothyroidism, and analyses from The Health Improvement Network database suggested an increased risk of stroke in individuals with positive TPOAb.^21,23^

Our findings build upon and extend a prior IPD meta-analysis by Collet et al.^20^, which found no difference in CHD risk by TPOAb status. This analysis was limited by older antibody assays in 2 included studies, a smaller sample size (38 274 participants vs. 99 287 in the current analysis for CHD outcomes), and did not include stroke outcomes. Most importantly, it did not adjust for residual confounding of TSH in its primary models, an established independent cardiovascular risk factor in thyroid dysfunction.^8,10^ By addressing these limitations, incorporating TSH adjustments, expanding the sample size, and including stroke outcomes, our findings provide the most robust observational evidence to date that positive TPOAb in subclinical hypothyroidism is not associated with an increased risk of CVD outcomes.

The increased risk of stroke we observed only in TPOAb-negative participants with subclinical hypothyroidism was contrary to our expectations. While levothyroxine treatment of TPOAb-positive participants, as recommended by some guidelines, may mitigate risk, we observed similar results in a sensitivity analysis when we excluded thyroid medication users who suggested otherwise. Previous studies have reported that positive TPOAb is independently associated with worse outcomes in ischemic stroke with no association reported for hemorrhagic stroke.^53,54^ A stratified analysis by stroke subtype would have aided the interpretation of our results, but several cohorts did not provide subtype information, and we were underpowered to run a stroke subtype analysis. Although it is plausible that positive TPOAb may be associated with an increased risk of ischemic stroke via atherosclerotic mechanisms, adequately powered studies are warranted to test this hypothesis and further explore the role of stroke subtype. Overall, our results should be interpreted with caution and not as evidence of the protective role of positive TPOAb. Given the limited biological plausibility for a reduced stroke risk among TPOAb-positive individuals, this association is likely a chance finding due to estimating multiple effects, though we cannot exclude residual confounding by unmeasured factors, such as differences in iodine status, C-reactive protein levels, and stroke subtype (ischemic stroke vs. hemorrhagic stroke).^55,56^

This study is the largest investigation to date into the association between TPOAb status and CHD risk in individuals with subclinical hypothyroidism and the first pooled analysis to explore stroke risk in this context. We used individual participant data from multiple studies (published and unpublished), with standardized definitions of thyroid measures and CVD outcomes. With this method, we were able to perform large-scale sensitivity analyses to investigate possible sources of heterogeneity and interactions by study-level and individual-level characteristics.

Nevertheless, our study also has some limitations which should be acknowledged. First, most cohorts included predominantly white populations from Europe and the United States, with the exception of 3 studies which were conducted in Iran and Brazil, limiting the generalizability of our findings to other populations and non-White individuals. Second, most studies measured thyroid function and TPOAb only at baseline, making it impossible to account for changes occurring over time in thyroid status or TPOAb status. However, findings from the Cardiovascular Health Study suggest that the risk of CHD remains consistent whether single or repeated TSH measurements are used, particularly among older adults.^57^ Furthermore, results from a recent IPD analysis by TSC, which included all age groups, showed that sensitivity analyses using repeated thyroid function measurements yielded similar CVD risk estimates, suggesting that potential misclassification is unlikely to have substantially influenced our findings.^58^ Finally, data were not available to account for stroke subtype analyses (ischemic vs. hemorrhagic) or C-reactive protein levels.

Our study has key clinical implications. Not finding evidence of an independent association between positive TPOAb and CHD and stroke outcomes before and after adjusting for TSH and traditional risk factors suggests that TPOAb testing is not warranted for cardiovascular risk stratification in the overall population or among individuals with subclinical hypothyroidism. Instead, our results reinforce the importance of the degree of thyroid dysfunction rather than a positive antibody alone in cardiovascular risk assessment and management strategies to optimize patient care for individuals with thyroid dysfunction.

## Conclusion

Positive TPOAb status was not associated with increased risk of CHD or stroke. These findings do not support the use of TPOAb testing for cardiovascular risk assessment in the overall population or among individuals with subclinical hypothyroidism. In individuals with subclinical hypothyroidism, risk stratification should focus on the degree of TSH elevation rather than antibody status alone.

## Supplementary Material

Supplementary Material

Supplementary material is available at European Journal of Endocrinology online.

## Figures and Tables

**Table 1.** Baseline characteristics of included cohorts and participants.

Study, year	Description of study sample	Country	*N*	Age^a^ at baseline median (range) year	Women, *N* (%)	Positive TPOAbs in overall, *N* (%)^b^	Subclinical hypothyroidism, *N* (%)	Subclinical hypothyroidism with positive TPOAbs, *N* (%)^c^	Thyroid medication at baseline, *N* (%)^d^	Thyroid medication during follow-up, *N* %	Follow-up time median (IQR) years^e^
ARIC Study, 1990	Adults aged 45–64 years	United States	12 676	57 (46–70)	6930 (54.7)	1388 (12.3)	736 (5.8)	350 (47.5)	381 (3.01)	580 (4.6)	22.80 (12.8–27.5)
Bari Study, 2006	Outpatients with stable chronic heart failure	Italy	642	65 (21–87)	138 (21.5)	59 (21.4)	34 (5.3)	10 (29.4)	43 (6.7)	42 (15.1)	4.10 (1.9–8.6)
Busselton Health Study, 1981	Community-dwelling adults in Busselton	Australia	2000	51 (18–89)	984 (49.2)	224 (11.2)	88 (4.4)	59 (67.1)	15 (0.75)	32 (1.6)	20 (14.1–20)
Cardiovascular Health Study, 1989	Community-dwelling adults	United States	3777	74 (64–88)	2196 (58.1)	489 (13.29)	516 (13.7)	198 (38.4)	265 (7.0)	591 (15.7)	10.1 (5.1–16.1)
Di@bet.es Study, 2009	Nationwide population	Spain	4381	49 (18–93)	2488 (56.8)	369 (8.40)	353 (8.1)	112 (31.1)	173 (3.9)	NA	10.3 (9.9–11.0)
ELSA-Brasil Study, 2008	Civil servants aged 35–74 years	Brazil	13 241	51 (35–74)	7121 (53.8)	1473 (11.2)	1043 (7.9)	310 (29.7)	813 (6.2)	1007 (8.2)	9.2 (8.7–9.5)
HUNT Study, 1995^g^	Adults living in Nord-Trøndelag	Norway	26 062	54 (20–97)	17 562 (67.4)	593 (34.2)	822 (3.2)	429 (52.2)	0 (0)	NA	12.3 (11.8–12.9)
Japanese Brazilian Thyroid Study, 1999	Adults of Japanese descent living in San Paulo	Brazil	982	56 (30–92)	518 (52.8)	86 (9.4)	92 (9.4)	26 (28.3)	0 (0)	NA	7.3 (7.1–7.5)
Leiden 85-plus Study, 1997	Adults aged 85 years	The Netherlands	482	85 (NA)	313 (64.9)	48 (11.4)	26 (5.4)	10 (38.5)	12 (2.5)	19 (3.9)	4.51 (2.1–5.0)
PREVEND Study, 1997	Adults in Groningen	The Netherlands	6324	52 (32–80)	3123 (49.4)	618 (9.8)	227 (3.6)	108 (47.6)	11 (0.2)	NA	13.9 (9.6–14.7)
Rotterdam	Adults ≥45 years living in Rotterdam	The Netherlands	8952	63 (45–106)	3920 (43.8)	1091 (12.2)	655 (7.3)	285 (43.5)	157 (3.4)	242 (4.1)	9.2 (7.3–15.0)
Study I-III, 1997											
SHIP Start, 1997^f^	Adults aged 20–79 years in West Pomerania	Germany	3830	49 (20–81)	1935 (50.5)	169 (9.0)	80 (2.1)	32 (40.0)	209 (5.5)	NA	13.7 (6.5–15.8)
Tehran Thyroid Study, 1997	Community-based adults aged ≥20 years	Iran	5143	30 (20–90)	2975 (57.9)	637 (12.4)	394 (7.7)	174 (44.2)	79 (1.6)	221 (4.8)	17.9 (13.9–18.5)
NHANES, 1999–2002 and 2007–2012	Noninstitutionalized adults	United States	11 758	47 (18–85)	5930 (50.5)	912 (10.5)	346 (2.9)	118 (34.1)	557 (4.75)	NA	7.7 (5.6–8.8)
Total			100 250	55 (18–106)	50 206 (56.7)	8155 (11.9)	5412 (5.4)	2221 (41.04)	2715 (2.9)	1695 (6.1)	12.1 (6.6–14.7)

Abbreviations: IQR, interquartile range (25th-75th percentiles); NA, data not available.

a Participants younger than 18 years were excluded.

b Overall *N* (%) for positive TPOAb was calculated based on the subset with available TPOAb data (*n* = 68 544), of whom 8155 were positive (11.9%).

c Number of participants with subclinical hypothyroidism and a positive TPOAb status.

d Data on thyroid medication use (T4, antithyroid drugs) were not available at follow-up for participants of the HUNT Study, the Japanese Brazilian Thyroid Study, NHANES, Di@bet.es Study 2009, and the PREVEND Study (NC1) 1997.

e For all cohorts, we used the maximal follow-up data that were available, which might differ from previous reports for some cohorts.

f Participants in the SHIP Start study had iodine supplementation a few years before inclusion; thus, a TSH reference range (0.25–2.12 mIU/L) was used as suggested.

g HUNT measured TPOAb only among participants with abnormal TSH and very few euthyroid participants, as reflected in the percentage.

**Table 2.** Association of TPOAb status (positive vs. negative) with CHD and stroke outcomes in the overall population.

	CHD events^a^				CHD mortality^a^			
	Positive TPOAb	Negative TPOAb	HR (95% CI) for positive vs. negative TPOAb	No. of studies	Positive TPOAb	Negative TPOAb	HR (95% CI) for positive vs. negative TPOAb	No. of studies
	Events/participants	Events/participants			Events/participants	Events/participants		
*Model 1*	674/4641	5333/34536	1.00 (0.90, 1.11)	10	274/7472	2310/58770	0.95 (0.78, 1.16)	13
*Model 2*	642/4277	5033/31379	0.99 (0.90, 1.10)	10	260/7028	2155/54709	0.96 (0.77, 1.21)	13
*Sex* ^b^								
Men	280/1774	3094/16947	1.01 (0.89, 1.14)	9	101/2664	1335/28807	0.86 (0.70, 1.07)	9
Women	394/2867	2239/17589	1.03 (0.82, 1.31)	9	173/4808	975/29963	1.15 (0.85, 1.55)	12
*P* interaction			*P* = .17				*P* = .56	
Age^c^								
<65 years	356/3269	2997/25052	1.00 (0.88, 1.12)	7	59/5501	725/44723	0.84 (0.63, 1.11)	8
≥65 years	318/1372	2336/9484	0.88 (0.67, 1.15)	10	215/1971	1585/14047	1.02 (0.87, 1.19)	11
*P* for interaction			*P* = .39				*P* = .11	
Excluding participants								
Excluding thyroid medication users at baseline^d^	562/3640	4940/28985	1.00 (0.85, 1.18)	10	220/5835	2055/52199	1.01 (0.84, 1.21)	12
Excluding thyroid medication users at baseline or during follow-up	392/2225	3782/18535	1.07 (0.89, 1.29)	6	123/2255	1342/18886	0.94 (0.64, 1.36)	6
	Stroke events^a^				Stroke mortality^a^			
*Model 1*	341/4082	2547/30920	0.98 (0.87, 1.11)	8	83/6602	608/51683	1.06 (0.81, 1.40)	10
*Model 2*	318/3744	2398/27933	0.96 (0.85, 1.08)	8	77/6186	572/47878	1.03 (0.78, 1.36)	10
*Sex* ^b^								
Men	128/1613	1255/15299	1.00 (0.82, 1.21)	8	34/2438	296/25582	1.19 (0.82, 1.72)	5
Women	213/2469	1292/15621	0.98 (0.84, 1.14)	8	49/4164	313/26101	1.08 (0.76, 1.54)	10
*P* for interaction			*P* = .48				*P* = .35	
*Age* ^c^								
<65 years	130/2677	1123/20931	0.95 (0.78, 1.15)	5	23/4703	203/38309	1.00 (0.63, 1.57)	5
≥65 years	211/1405	1424/9989	1.02 (0.88, 1.19)	8	60/1899	406/13374	1.10 (0.81, 1.50)	10
*P* for interaction			*P* = .52				*P* = .53	
Excluding participants								
Excluding thyroid medication users at baseline^d^	277/3095	2279/25338	0.99 (0.87, 1.13)	8	62/5041	524/45274	1.04 (0.79, 1.38)	10
Excluding thyroid medication users at baseline and during follow-up	191/1786	1649/15313	1.03 (0.88, 1.21)	4	35/1789	305/15337	1.07 (0.55, 2.09)	4

Abbreviations: CI, confidence interval; CHD, coronary heart disease; TPOAb, antithyroid peroxidase antibodies. HR, hazard ratio. HR > 1 indicates a higher hazard in the positive TPOAb group.

Model 1 is adjusted for age, sex, and log TSH. Model 2 is adjusted for age, sex, log TSH, BMI, systolic blood pressure, smoking, total cholesterol, and diabetes at baseline.

a The HUNT study was excluded from the analysis because it measured TPOAb only among participants with abnormal TSH.

b Age- and TSH (log scale)-adjusted.

c These HRs were adjusted for log TSH, sex, and age as continuous variable to avoid residual confounding within age strata.

d In some strata, specific studies were excluded from the analysis due to the absence of events in the comparison group.

**Table 3.** Association between TPOAb status and CHD outcomes in subclinical hypothyroidism.

	Euthyroid Events/participants	SH with positive TPOAb Status Events/participants	SH with negative TPOAb status Events/participants	SH with positive TPOAb vs. euthyroid HR (95% CI)	SH with negative TPOAb vs. euthyroid HR (95% CI)	SH with positive TPOAb vs. SH with negative TPOAb HR (95% CI)	*P*-value for HR positive vs. negative and no. of studies
**CHD events**							
Overall^a^	7341/65191	210/1627	297/1853	1.02 (0.85, 1.22)	1.13 (0.97, 1.32)	0.87 (0.72, 1.05)	*P* = .14; *n* = 9
*Sex* ^b^							
Men	4188/27884	78/517	140/743	0.95 (0.71, 1.28)	1.10 (0.87, 1.38)	0.93 (0.69, 1.27)	*P* = .65; *n* = 8
Women	3153/37307	132/1110	157/1110	1.22 (0.87, 1.71)	1.24 (0.99, 1.52)	0.91 (0.63, 1.32)	*P* = .53; *n* = 9
*P* **for interaction**				*P* = .34	*P* = .14		
*Age* ^c^							
<65 years	4009/47837	84/1047	105/1087	1.06 (0.79, 1.43)	1.14 (0.88, 1.47)	0.91 (0.59, 1.39)	*P* = .66; *n* = 5
≥65 years	3332/17354	126/580	192/766	1.00 (0.78, 1.29)	1.16 (0.94, 1.42)	0.82 (0.57, 1.18)	*P* = .28; *n* = 8
***P*** interaction				*P* = .31	*P* = .43		
TSH Category^d^							
Euthyroid				1 (reference)	1 (reference)		
4.5–6.9 mIU/L	7341/65191	122/996	245/1512	0.97 (0.81, 1.16)	1.09 (0.96, 1.24)	0.85 (0.68, 1.07)	*P* = .16; *n* = 9
7.0–9.9 mIU/L	7265/64737	50/390	35/248	0.92 (0.70, 1.22)	1.12 (0.70, 1.80)	0.94 (0.59, 1.51)	*P* = .81; *n* = 6
10.0–19.9 mIU/L	7265/64737	38/241	17/93	1.25 (0.87, 1.80)	1.67 (1.03, 2.69)	1.00 (0.49, 2.01)	*P* = .99; *n* = 5
***P*** for trend				*P* = .22	*P* = .07		
**CHD mortality**							
Overall^a^	3122/93984	93/2188	173/3115	1.18 (0.81, 1.72)	1.23 (1.03, 1.48)	0.88 (0.64, 1.21)	*P* = .43; *n* = 9
*Sex* ^b^							
Men	1728/41580	35/682	99/1258	1.13 (0.77, 1.65)	1.46 (1.14, 1.86)	0.77 (0.50, 1.19)	*P* = .24; *n* = 7
Women	1394/52404	58/1506	70/1775	1.61 (0.87, 3.00)	1.09 (0.77, 1.56)	1.21 (0.57, 2.54)	*P* = .64; *n* = 7
*P* **for interaction**				*P* = .65	*P* = .01		
*Age* ^c^							
<65 years	949/71124	11/1489	24/2007	0.84 (0.39, 1.82)	2.49 (0.92, 6.70)	0.52 (0.20, 1.32)	*P* = .17; *n* = 2
≥65 years	2173/22860	149/1108	82/699	1.31 (1.03, 1.66)	1.38 (0.83, 2.30)	0.90 (0.65, 1.26)	*P* = .55; *n* = 9
***P*** interaction				*P* = .27	*P* = .93		
TSH Category^d^							
Euthyroid				1 (reference)	1 (reference)		
4.5–6.9 mIU/L	3122/93984	53/1369	145/2536	1.17 (0.73, 1.87)	1.18 (0.95, 1.46)	0.86 (0.56, 1.31)	*P* = .47; *n* = 8
7.0–9.9 mIU/L	3080/93533	22/519	17/423	0.95 (0.62, 1.45)	1.10 (0.63, 1.92)	1.34 (0.63, 2.85)	*P* = .45; *n* = 6
10.0–19.9 mIU/L	3019/90377	18/300	11/156	1.80 (1.13, 2.87)	1.60 (0.88, 2.90)	0.90 (0.33, 2.46)	*P* = .84; *n* = 2
***P*** for trend				*P* = .28	*P* = .19		

Abbreviations: CI, confidence interval; CHD, coronary heart disease; HR, hazard ratio; SH, subclinical hypothyroidism; TPOAb, antithyroid peroxidase antibodies; HR > 1 indicates a higher hazard in the positive TPOAb group.

a Adjusted for age, sex, and TSH (log scale).

b Adjusted for age and TSH (log scale).

c Adjusted for TSH (log scale) and sex and age as continuous variables to avoid residual confounding within age strata.

d In some strata, specific studies were excluded from the analysis due to the absence of events in the comparison group.

**Table 4.** Association between TPOAb status and stroke outcomes in subclinical hypothyroidism.

	Euthyroid Events/participants	SH with positive TPOAb status Events/participants	SH With negative TPOAb status Events/participants	SH with positive TPOAb vs. euthyroid HR (95% CI)	SH with negative TPOAb vs. euthyroid HR (95% CI)	SH with positive TPOAb vs. SH with negative TPOAb HR (95% CI)	*P*-value for HR positive vs. negative and no. of studies
**Stroke events**							
Overall^a^	2941/36118	83/1052	153/1315	0.94 (0.70, 1.23)	1.24 (1.03, 1.55)	0.68 (0.51, 0.90)	*P* = .01; *n* = 5
Sex^b^							
Men	1452/17834	29/414	70/616	0.94 (0.63, 1.43)	1.32 (0.97, 1.81)	0.65 (0.41, 1.05)	*P* = .07; *n* = 4
Women	1489/18284	54/638	83/699	0.92 (0.67, 1.25)	1.18 (0.91, 1.53)	0.73 (0.51, 1.05)	*P* = .09; *n* = 5
***P*** interaction				*P* = .81	*P* = .73		
Age^c^							
<65 years	1367/25033	25/616	47/670	1.00 (0.65, 1.54)	1.39 (1.01, 1.93)	0.60 (0.35, 1.03)	*P* = .07; *n* = 3
≥65 years	1574/11085	58/436	106/645	0.88 (0.65, 1.19)	1.19 (0.94, 1.50)	0.71 (0.50, 1.02)	*P* = .06; *n* = 5
***P*** interaction				*P* = .19	*P* = .04		
TSH Category^d^							
Euthyroid				1 (reference)	1 (reference)		
4.5–6.9 mIU/L	2941/36118	47/640	119/1073	0.77 (0.58, 1.03)	1.09 (0.91, 1.31)	0.73 (0.51, 1.03)	*P* = .07; *n = 5*
7.0–9.9 mIU/L	2934/35510	20/242	24/169	0.77 (0.50, 1.20)	1.36 (0.78, 2.04)	0.52 (0.27, 1.02)	*P* = .06; *n* = 4
10.0–19.9 mIU/L	2882/35664	16/170	10/73	1.03 (0.63, 1.68)	1.70 (0.91, 3.16)	0.60 (0.23, 1.56)	*P* = .30; *n* = 3
***P*** for trend				*P* = .22	*P* = .16		
**Stroke mortality**							
Overall^a^	757/61465	18/1516	30/2362	1.14 (0.67, 1.93)	1.33 (0.87, 2.04)	0.93 (0.51, 1.90)	*P* = .28; *n* = 6
Sex^b^							
Men	369/30014	4/559	16/1128	0.93 (0.26, 3.31)	1.35 (0.74, 2.48)	0.60 (0.18, 1.94)	*P* = .36; *n* = 2
Women	388/31451	14/957	14/1234	1.84 (0.94, 3.61)	1.86 (0.98, 3.51)	1.02 (0.40, 2.61)	*P* = .81; *n* = 4
***P*** for interaction				*P* = 0.98	*P* = 0.74		
Age^c^							
<65 years	253/46021	5/989	9/1463	2.45 (0.71, 8.41)	1.71 (0.64, 4.57)	0.89 (0.24, 3.25)	*P* = .86; *n* = 2
≥65 years	504/15444	13/527	21/899	0.86 (0.49, 1.50)	1.05 (0.67, 1.64)	0.60 (0.27, 1.34)	*P* = .22; *n* = 4
***P*** interaction				*P* = .24	*P* = .21		
TSH Category^d^							
Euthyroid				1 (reference)	1 (reference)		
4.5–6.9 mIU/L	757/61465	11/950	22/1923	0.92 (0.51, 1.68)	0.89 (0.58, 1.37)	0.87 (0.38, 1.97)	*P* = .73; *n* = 5
7.0–9.9 mIU/L	756/60857	5/352	5/318	1.78 (0.42, 7.59)	1.49 (0.61, 3.63)	0.47 (0.08, 2.78)	*P* = .40; *n* = 3
10.0–19.9 mIU/L	711/61014	2/214	3/121	1.97 (0.24, 3.91)	4.43 (1.38, 14.15)	0.69 (0.05, 8.95)	*P* = .78; *n* = 2
***P*** for trend				*P* = .09	*P* = .07		

Abbreviations: CI, confidence interval; CHD, coronary heart disease; HR, hazard ratio; SH, subclinical hypothyroidism; TPOAb, antithyroid peroxidase antibodies. HR > 1 indicates a higher hazard in the positive TPOAb group.

a Adjusted for age, sex, and TSH (log scale).

b Adjusted for age and TSH (log scale).

c Adjusted for TSH (log scale) and sex and age as continuous variables to avoid residual confounding within age strata.

d In some strata, specific studies were excluded from the analysis due to the absence of events in the comparison group.

## Data Availability

Due to data protection agreements with the TSC and participating cohorts, individual-level data from these cohorts cannot be shared with third parties.

## Significance

A positive test for TPOAb is frequently detected in thyroid dysfunction and is commonly measured in the clinical setting. Whether a positive TPOAB status is independently associated with cardiovascular risk beyond thyroid function remains uncertain. Using individual participant data from over 100 000 adults across 14 cohorts with long-term follow-up, our study is the largest and most comprehensive analysis to date addressing this question. We found that a positive TPOAb test is not associated with an increased risk of coronary heart disease or stroke, independent of thyroid function or traditional cardiovascular risk factors. These findings have important clinical implications showing that TPOAb testing adds no value to cardiovascular risk assessment and reinforcing the importance of the degree of thyroid dysfunction itself in guiding clinical management.
