# Supplementary material for: Thyroid antibody status, thyroid function, and the risk of coronary heart disease and stroke: an individual participant data analysis from 14 cohorts
Source: Eur J Endocrinol. Author manuscript; Available in PMC 2026 May 25. (PMC13200528; doi:10.1093/ejendo/lvaf209)
Supplement: Supplementary Material [file NIHMS2177077-supplement-Supplementary_Material.docx]

**Supplementary**

**eFigure 1.** PRISMA Flow Diagram of Study Selection

**eTable 1.** Definitions of Subclinical Hypothyroidism, Thyroid Antibody Assays, CHD and Stroke Events, and Mortality

**eFigure 2.** Forest Plot of Hazard Ratios for CHD Events in All Participants

**eFigure 3.** Forest Plot of Hazard Ratios for CHD Mortality in All Participants

**eFigure 4.** Forest Plot of Hazard Ratios for Stroke Events in All Participants

**eFigure 5.** Forest Plot of Hazard Ratios for Stroke Mortality in All Participants

**eTable 2.** Association Between TPOAb Status and CHD and Stroke Outcomes in Euthyroid Participants

**eTable 3.** Sensitivity Analyses: Association Between TPOAb Status with CHD Outcomes in Subclinical Hypothyroidism

**eTable 4.** Sensitivity Analyses: Association Between TPOAb Status with Stroke Outcomes in Subclinical Hypothyroidism

**eTable 5.** Quality Assessment of Included Cohorts Using the Newcastle-Ottawa Scale

**eFigure 6.** The proportional hazards assumption of the Cox model assessed at the study level with graphical methods based on Schoenfeld residuals.

**eFigure 7.** PRISMA Checklist (Preferred Reporting Items for Systematic Reviews and Meta-Analyses)

**eMethods**

**Search strategy**

**Embase.com**

('subclinical hypothyroidism'/de OR 'hypothyroidism'/de OR 'thyroid function'/exp OR 'thyroid function test'/de OR 'thyroid disease'/de OR 'thyrotropin'/de OR 'thyrotropin blood level'/de OR 'thyroid hormone'/de OR 'thyroid hormone blood level'/exp OR 'thyroid peroxidase antibody'/exp OR 'thyroglobulin antibody'/de OR 'euthyroid sick syndrome'/de OR ((thyroid* NEAR/3 (function* OR dysfunction* OR disorder* OR disease* OR failure* OR autoimmun* OR auto-immun* OR hormone* OR autoantibod* OR antibod*)) OR (antithyroid NEAR/2 antibod*) OR thyroidit* OR hypothyro* OR thyrotropin* OR TSH OR l-thyroxin* OR levothyroxine* OR eltroxin* OR levoxyl OR synthroid OR unithroid OR tetraiodothyronin* OR ((t4 OR ft4 OR t-4 OR ft-4 OR t3 OR ft3 OR ft-3 OR TSH OR thyreotropin OR thyroxin*) NEAR/3 (free OR plasma OR blood OR serum OR level* OR concentrat* OR low OR high OR elevat* OR deficien* OR decrease* OR increase* OR 'normal range')) OR (thyroid* NEAR/3 peroxidase* NEAR/3 antibod*) OR ((tpo OR thyroglobulin* OR thyroperoxidas* OR thyroperoxid*) NEAR/3 (antibod* OR positiv* OR negativ* OR status*)) OR (euthyroid* OR eu-thyroid*)):ti,ab,kw) AND AND ('cardiovascular disease'/de OR 'heart disease'/exp OR 'mortality'/de OR 'cardiovascular mortality'/de OR 'mortality rate'/de OR 'survival'/exp OR 'survival rate'/de OR 'cardiovascular risk'/de OR (((cardiovascular* OR cv OR cvd OR heart OR cardiac OR myocard* OR atrial OR atrium) NEAR/3 (disease* OR disorder* OR failure OR ischemi* OR ischaemi* * OR risk OR infarct* OR morbid* OR event*)) OR mortalit* OR survival):ab,ti) AND ('cohort analysis'/exp OR 'longitudinal study'/de OR 'prospective study'/de OR 'controlled clinical trial'/exp OR 'epidemiology'/de OR 'incidence'/de OR 'follow up'/de OR 'population research'/de OR (cohort* OR longitudinal* OR prospectiv* OR (controlled NEAR/6 (clinical OR random*) NEAR/6 trial*) OR follow-up* OR followup* OR incidence* OR (population NEAR/3 (sample* OR research OR general))):ab,ti) NOT ((child/exp OR pediatrics/exp OR (child* OR pediatr* OR infan*):ab,ti) NOT (adult/exp OR (adult* OR elder*):ab,ti)) NOT ([animals]/lim NOT [humans]/lim)

**MEDLINE (Ovid)**

(Hypothyroidism/ OR Thyroid Diseases/ OR Thyroid Function Tests/ OR Thyrotropin/ OR Thyroxine/ OR exp Thyroid Hormones/ OR Euthyroid Sick Syndromes/ OR ((thyroid* adj3 (function* OR dysfunction* OR disORder* OR disease* OR failure* OR autoimmun* OR auto-immun* OR hORmone* OR autoantibod* OR antibod*)) OR (antithyroid adj2 antibod*) OR thyroidit* OR hypothyro* OR thyrotropin* OR tsh OR l-thyroxin* OR levothyroxine* OR eltroxin* OR levoxyl OR synthroid OR unithroid OR tetraiodothyronin* OR ((t4 OR ft4 OR t-4 OR ft-4 OR t3 OR ft3 OR ft-3 OR tsh OR thyreotropin OR thyroxin*) adj3 (free OR plasma OR blood OR serum OR level* OR concentrat* OR low OR high OR elevat* OR deficienc* OR decrease* OR increase* OR normal range)) OR (thyroid* adj3 peroxidase* adj3 antibod*) OR ((tpo OR thyroglobulin* OR thyroperoxidas* OR thyroperoxid*) adj3 (antibod* OR positiv* OR negativ* OR status*)) OR euthyroid* OR eu-thyroid*).ti,ab,kf.) AND ("Cardiovascular Diseases"/ OR exp "Heart Diseases"/ OR exp "Mortality"/ OR "Fatal Outcome"/ OR mortality.xs. OR "survival"/ OR "survival rate"/ OR (((cardiovascular* OR cv OR cvd OR heart OR cardiac OR myocard* OR atrial OR atrium) ADJ3 (disease* OR disorder* OR failure OR ischemi* OR ischaemi* OR risk OR infarct* OR morbid* OR event*)) OR mortalit* OR survival).ab,ti,kf.) AND (exp "Cohort Studies"/ OR exp "controlled clinical trial"/ OR "epidemiology"/ OR "incidence"/ OR (cohort* OR longitudinal* OR prospectiv* OR (controlled ADJ6 (clinical OR random*) ADJ6 trial*) OR follow-up* OR followup* OR incidence* OR (population ADJ3 (sample* OR research OR general))).ab,ti,kf.) NOT ((exp child/ OR exp infant/ OR exp pediatrics/ OR (child* OR pediatr* OR infan*).ab,ti,kf.) NOT (exp adult/ OR (adult* OR elder*).ab,ti,kf.)) NOT (exp animals/ NOT humans/)

**Cochrane CENTRAL**

(((thyroid* NEAR/3 (function* OR dysfunction* OR disorder* OR disease* OR failure* OR autoimmun* OR auto-immun* OR hormone* OR autoantibod* OR antibod*)) OR (antithyroid NEAR/2 antibod*) OR thyroidit* OR hypothyro* OR thyrotropin* OR TSH OR l-thyroxin* OR levothyroxine* OR eltroxin* OR levoxyl OR synthroid OR unithroid OR tetraiodothyronin* OR ((t4 OR ft4 OR t-4 OR ft-4 OR t3 OR ft3 OR ft-3 OR TSH OR thyreotropin OR thyroxin*) NEAR/3 (free OR plasma OR blood OR serum OR level* OR concentrat* OR low OR high OR elevat* OR deficien* OR decrease* OR increase* OR "normal range")) OR (thyroid* NEAR/3 peroxidase* NEAR/3 antibod*) OR ((tpo OR thyroglobulin* OR thyroperoxidas* OR thyroperoxid*) NEAR/3 (antibod* OR positiv* OR negativ* OR status*)) OR (euthyroid* OR eu-thyroid*)):ti,ab,kw) AND ((((cardiovascular* OR cv OR cvd OR heart OR cardiac OR myocard* OR atrial OR atrium) NEAR/3 (disease* OR disorder* OR failure OR ischemi* OR ischaemi* OR arrythm* OR fibrillat* OR flutter* OR risk OR infarct* OR morbid* OR event*)) OR mortalit* OR survival):ab,ti) AND ((cohort* OR longitudinal* OR prospectiv* OR (controlled NEAR/6 (clinical OR random*) NEAR/6 trial*) OR follow-up* OR followup* OR incidence* OR (population NEAR/3 (sample* OR research OR general))):ab,ti) NOT (((child* OR pediatr* OR infan*):ab,ti) NOT ((adult* OR elder*):ab,ti))

**eFigure 1. Adapted from PRISMA Flow Chart**

Additional records identified through TSC
(n = 9)

Records identified through database searching
(n = 4713)

Records after duplicates removed
(n = 3935)

**Studies included in
IPD-analysis**
(n = 14)

Studies included after full-text screening
(n = 19)

Records screened
(n = 3935)

Records excluded based on title and abstract
(n = 3813)

Full-text articles excluded (n = 103)
- Publication from same cohort (N=59)
- Specific patient populations (N=34)
- No CVD outcomes available (N=8)
- Retrospective cohort study (N=2)

Full-text articles assessed for eligibility
(n = 122)

Excluded from IPD (5)

- No response (N=3)
- Data not available or eligible (N=2)

Fourteen cohorts were identified through a systematic literature search conducted in July 2023 and updated in October 2024.

**eTable 1. Definition of subclinical hypothyroidism, thyroid antibody assay, CHD and stroke events and mortality**

| **Study** | **Subclinical hypothyroidism definition** | **Thyroid antibody assay – Description and cutoff** | **TSH assays** | **Event definition^g^** | |
| --- | --- | --- | --- | --- | --- |
|  |  |  |  | **CHD events and mortality – Methods for ascertainment** | **Stroke events and mortality** |
| ARIC Study 1990 | TSH ≥ 4.5 mIU/L & TSH < 20, normal FT4 0.85–1.4 ng/dL (11-18 pmol/L) pmol/L | Anti-thyroid peroxidase antibodies Roche e411 Immunoassay Analyzer (Roche Diagnostics Corporation, Indianapolis, IN; Positive if ≥34 IU/ml | Roche Diagnostics, Elecsys 2010 Analyzer, Advanced Research Diagnostics Laboratory (University of Minnesota) | CHD mortality includes a definite or probable fatal MI (i.e., expert-adjudicated definite or probable MI followed by death within 28 days) or CHD death (i.e., death from MI not meeting the criteria for definite or probable, or sudden death preceded by cardiac symptoms or signs without evidence of non-coronary causes [e.g., stroke])  CHD includes a definite or probable nonfatal MI and CHD mortality | Stroke mortality (ICD-9 codes 430 to 438)  Stroke events include hospitalization and death from stroke (ICD-9 430–437; ICD-10 I60–68 G45), and peripheral arterial disease (ICD-9 440–448; ICD-10 I70–79). |
| Bari Study 2006 | TSH ≥ 4.5mIU/L & TSH < 20mIU/L normal fT4 0.7-1.8 ng/dL (9-23 pmol/L) | Anti-thyroid peroxidase antibodies (AutoDelfia assay, PerkinElmer Life and Analytical Sciences, Wallac Oy); Positive if ≥ 35 IU/mL | Immunoassay, Advia Centaur, Bayer Diagnostics Division, Tarrytown, NY | CHD mortality is defined as death due to sudden death and coronary heart disease.  CHD events (MI, angina pectoris, acute coronary syndrome, percutaneous transluminal coronary angioplasty), cardiovascular causes for CHD mortality | Stroke mortality is death due to stroke  Stroke events hospitalization with stroke and stroke mortality |
| Busselton Health Study 1981 | TSH ≥ 4.5 mIU/L & TSH < mIU/L, normal FT4 9-23 pmol/L or missing FT4 (1/89, 1.1%) | Anti-thyroid peroxidase antibodies (Immulite 2000 chemiluminescent analyzer, Seimans Healthcare Diagnostics, Deerfield, IL); Positive if ≥ 35 IU/mL | Immulite 2000 chemiluminescent analyzer (Diagnostic Products Corporation, Los Angeles, Calif.) | CHD mortality (ICD9 410-414). Ascertained from death records.  CHD events, such as death from cardiovascular disease and principal discharge diagnosis of CHD (ICD-9 410–414; ICD-10 I20–25), | First stroke event and death from stroke ICD-9 codes 430–438 (& ICD-10 I60-I69 including G45) |
| Cardiovascular Health Study 1989 | TSH ≥4.5 mIU/L & TSH < 20 mIU/L  normal fT4 0.7-1.7 ng/dL (9-22 pmo1/L) or missing fT4 (0/543, 4.2%) | Anti-thyroid peroxidase antibodies (chemiluminescent immunoassays on the Elecsys 2010 analyzer; Roche Diagnostics, Indianapolis, IN); Positive if ≥ 37 IU/mL | LumaTaghTSH chemiluminescence (Nichols Institute, San Juan Capistrano, CA) | Medical records, death certificates, autopsy reports, and coroners’ reports reviewed by experts CHD mortality as death due to atherosclerosis (including peripheral vascular disease), coronary heart disease and other cardiovascular causes.  CHD events as MI, angina, coronary angioplasty, coronary artery surgery, atherosclerotic CHD mortality | Stroke mortality (ICD-9 430–437).  Stroke events include hospitalization and death from stroke all were adjudicated by CHS Events Subcommittee. |
| Di@bet.es Study 2009 | TSH ≥ 4.5mIU/L & TSH < 20mIU/L normal fT4 11- 22pmol/L) | Anti-thyroid peroxidase antibodies (Modular Analytics E170, cobas e 602; Roche Diagnostics, Basel, Switzerland)); Positive if ≥ 50 IU/mL | Electrochemiluminescence immunoassay (Modular Analytics E170, cobas e 602; Roche Diagnostics, Basel, Switzerland) | Non-fatal AMI 410.xx Non-fatal stroke 346.6; 431.xx; 432.xx; 432.9; 433.x1; 434.x1; 435.xx; 436.xx; 437.1 Unstable angina 413.xx Heart failure 398.91; 404.11; 404.13; 411.89; 433.x1; 428.xx Revascularisation 00.66; 17.55; 36.x | Stroke mortality (ICD-9 430–437).  Stroke events (ICD-9 346.6 and 430–437) |
| ELSA-Brasil Study 2008 | TSH ≥ 4.5mIU/L & TSH < 20mIU/L normal fT4 0.7-1.7ng/dL (9- 22pmol/L) | Anti-thyroid peroxidase antibodies (electrochemiluminescence (Roche Diagnostics, Mannheim, Germany); Positive if ≥ 34IU/mL | Third-generation immunoenzymatic assay (Roche Diagnostics, Manheim, Germany) | CHD mortality ICD-10 I00-I99 obtained from hospital admissions records, death certificates or via cross-checking with data from the mortality system of the Ministry of Health.  CHD events NA | Stroke mortality ICD-10 I60–68 G45),  Stroke events NA |
| HUNT Study 1995 | TSH ≥ 4.5mIU/L & TSH < 20mIU/L  normal fT4 0.6-1.6ng/dL (8- 20pmol/L) or missing fT4 (0/814, 0%) | Anti-thyroid peroxidase antibodies (Lumino- immunoassay, Brahms Diagnostica GmbH, Berlin, Germany); Measured if TSH >4.0 mIU/L; Positive if > 200 IU/mL | DELFIA hTSH Ultra | CHD mortality / Death certificates; causes of death coded with ICD9 410-414; ICD10 I20-I25  CHD events as hospitalization for MI, CHD mortality | NA |
| Japanese Brazilian Thyroid Study 1999 | TSH ≥ 4.5 mIU/L & TSH < 20  mIU/L, normal FT4 0.7-1.5 ng/dL (no missing FT4) | Anti-thyroid peroxidase antibodies (AutoDelfia assay, PerkinElmer Life and Analytical Sciences, Wallac Oy); Positive if ≥ 35 IU/mL | Sensitive immunofluorometric assay (Wallac–Delfia, PerkinElmer, Turku, Finland) | CHD mortality as death from any cardiovascular event obtained from death certificates.  CHD events NA | Stroke mortality as death from any cerebrovascular event obtained from death certificates.  Stroke events NA |
| Leiden 85-plus Study 1997 | TSH ≥ 4.5mIU/L & TSH < 20mIU/Lnormal fT4 0.6-1.6ng/dL (13- 23pmol/L) or missing fT4 (0/814, 0%) | Anti-thyroid peroxidase antibodies (Immulite 2000 XPi Immunoassay System, Siemens AG, Berlin, Germany) ); Positive if ≥ 35 IU/mL | Elecsys 2010 system + electrochemiluminescence (Boehringer, Germany) | Death from ischaemic heart diseases (ICD-10 I20-I25) and stroke (ICD-10 I60-I69).  CHD events MI + CHD mortality defined based on the data from primary care physician, ECGs and death registry forms. | Stroke mortality as (ICD-10 I60-I69).  Stroke events defined based on the data from primary care physician, ECGs and death registry forms. |
| PREVEND Study 1997 | (NC1) TSH ≥ 4.5 mIU/L & TSH < 20mIU/L, normal 9.14–23.81 pmol/L (no missing FT4)  (NC2) TSH ≥ 4.5 mIU/L & TSH < 20mIU/L, normal 12-22 pmol/L (no missing FT4) | Anti-thyroid peroxidase antibodies (AutoDelfia assay, PerkinElmer Life and Analytical Sciences, Wallac Oy); Positive if ≥ 35 IU/mL | (NC1) Architect; Abbott Laboratories, Abbott Park, IL, USA.  (NC2) Roche Modular E170 Analyzer electrochemiluminescent immunoassays (Roche Diagnostics, Mannheim, Germany) | CVD mortality. ICD-10 I10-I90. Records were obtained from municipality records and classified by a physician from Statistics Netherlands.  CHD event defined as the combined endpoint of acute myocardial infarction, acute and subacute ischemic heart disease, coronary artery bypass grafting or percutaneous transluminal coronary angioplasty and CHd mortality reviewed by endpoint adjudication committee. | Stroke mortality as death from stroke  Stroke event defined as subarachnoid haemorrhage, intracerebral haemorrhage, other intracranial haemorrhage, occlusion or stenosis of the precerebral or cerebral arteries, and death from stroke reviewed by endpoint adjudication committee. |
| Rotterdam Study I-III 1997 | TSH ≥ 4.5 mIU/L & TSH < 20mIU/L, normal 12-22 pmol/L (no missing FT4) | Positive if ≥ 35 IU/mL | Electrochemiluminescence immunoassay for thyroxine and thyrotropin, ECLIA, Roche | CVD mortality is defined as definite fatal MI, definite fatal CHD, and possible fatal CHD, other atherosclerotic disease, and other CVD. Causes of death were obtained from general practitioners or hospital records and were coded independently by two physicians and subsequently validated by a medical specialist.  CHD event was defined as fatal or non-fatal MI, percutaneous coronary interventions, coronary artery bypass graft or cardiovascular mortality. | Stroke death as death from nontraumatic cerebrovascular disease.  Stroke event was defined as a syndrome of rapidly developing symptoms, with an apparent vascular cause of focal or global cerebral dysfunction lasting 24 hours or longer or leading to death. in accordance with WHO criteria. Cases were ascertained through a combination of routine in-person assessment and medical records, with final diagnosis made by a consensus panel led by a consultant neurologist. |
| SHIP Start Study 1997^b^ | TSH ≥ 2.12 mIU/L & TSH< 17.6 mIU/L ^‡^, normal FT4 8.3-18.9 pmol/L (no missing FT4) | Anti-thyroid peroxidase antibodies (VARELISA, Elias Medizintechnik GmbH, Freiburg, Germany); Positive if > 200 IU/mL | Baseline: immunochemiluminescentprocedure (LIA-mat, Byk Sangtec Diagnostica GmbH,Frankfurt, Germany) . Follow-up: (Immulite 2000, Third Generation,Diagnostic Products Corporation (DPC), Los Angeles,CA, USA) | Death certificates from local health authority; independent validation of cause of death; review with another expert if disagreement; coded with ICD10 I20-I25  CHD events as non-fatal MI Self-reported physician diagnosis of stroke and myocardial infarction and self-reported heart surgery and CHd mortality | Stroke mortality as death certificates from local health authority; independent validation of cause of death; review with another expert if disagreement; coded with  ICD-10 I10-I79  Stroke events as Self-reported physician diagnosis of stroke |
| Tehran Thyroid Study 1997 | TSH ≥ 4.5mIU/L & TSH < 20mIU/L normal fT4 0.9-1.9ng/dL (12-20pmol/L) or missing fT4 (35/107, 32.7%) | Anti-thyroid peroxidase (Roche Diagnostic’s kits & Roche/Hitachi Cobas e-411 analyser (GmbH, Mannheim, Germany).) Positive if >35 kU/L | Electrochemiluminescence immunoassay (ECLIA) method, using Roche Diagnostic’s kits & Roche/Hitachi Cobas e-411 analyzer (GmbH, Mannheim, Germany). | CHD events included cases of definite MI diagnosed by ECG and biomarkers (creatine phosphokinase-MB, lactate dehydrogenase, troponin), probable MI (positive ECG findings plus cardiac symptoms or signs and biomarkers showing negative or equivocal results), unstable angina pectoris (new cardiac symptoms or changing symptom patterns and positive ECG findings with normal biomarkers), angiographic-proven CHD and cardiac death. Death from CHD obtained from the death certificate or medical records confirmed by an outcome committee. | NA |
| NHANES 1999-2002 2007-2012 | TSH ≥ 4.5mIU/L & TSH < 20mIU/L ^‡^, normal FT4 0.6-1.6 8.3-18.9 pmol/L (no missing FT4) | Anti-thyroid peroxidase (chemiluminescence immunometric assay) Positive if ≥ 9 kU/L | chemiluminescence immunometric assay (Nichols Institute Diagnostics, San Juan Capistrano, CA) | CHD mortality as ICD-10 I00-I09, I11, I13, I20-I51 obtained through the National Death Index.  CHD events NA | Stroke mortality ICD-10 I60-I69 obtained through the National Death Index.  Stroke events NA |

**eFigure 2. Forest plots for CHD events in all participants**

**B) CHD events of all Participants according to TPOAb status at Baseline (positive vs. negative) Adjusted for TSH, age and sex, smoking status, BMI, systolic blood pressure. diabetes mellitus, and total cholesterol**

**A) CHD events of all Participants according to TPOAb status at Baseline (positive vs. negative) Adjusted for TSH, age and sex.**

**eFigure 3. Forest plots for CHD mortality in all participants**

**B) CHD Mortality of all Participants according to TPOAb status at Baseline (positive vs. negative) Adjusted for TSH, age and sex, smoking status, BMI, systolic blood pressure. diabetes mellitus, and total cholesterol**

**A) CHD Mortality of all Participants according to TPOAb status at Baseline (positive vs. negative) Adjusted for TSH, age and sex.**

**eFigure 4. Forest plots for Stroke events in all participants**

**B) Stroke events of all Participants according to TPOAb status at Baseline (positive vs. negative) Adjusted for TSH, age and sex, smoking status, BMI, systolic blood pressure. diabetes mellitus, and total cholesterol**

**A) Stroke events of all Participants according to TPOAb status at Baseline (positive vs. negative) Adjusted for TSH, age and sex.**

**eFigure 5. Forest plots for Stroke mortality in all participants**

**A) CHD events of all Participants according to TPOAb status at Baseline (positive vs. negative) Adjusted for TSH, age and sex.**

**B) Stroke events of all Participants according to TPOAb status at Baseline (positive vs. negative) Adjusted for TSH, age and sex, smoking status, BMI, systolic blood pressure. diabetes mellitus, and total cholesterol**

**eTable2. Association Between TPOAb Status and CHD and Stroke Outcomes in Euthyroid Participants, Adjusted for Age and Sex.**

|  | CHD Events ^a^ | | | | CHD mortality ^a^ | | | |
| --- | --- | --- | --- | --- | --- | --- | --- | --- |
|  | **Positive**  **TPOAb** | **Negative**  **TPOAb** | **HR (95% CI) for positive vs. negative TPOAb** | **No. of studies** | **Positive**  **TPOAb** | **Negative**  **TPOAb** | **HR (95% CI) for positive vs. negative TPOAb** | No. of  studies |
|  | **Events/**  **Participants** | **Events/**  **Participants** |  |  | **Events/**  **Participants** | **Events/**  **Participants** |  |  |
| *Model 1* | 703/5234 | 5415/35675 | 1.02 (0.89, 1.18) | 11 | 274/7472 | 2310/58770 | 0.95 (0.78, 1.16) | 13 |
| *Sex*^†^ |  |  |  |  |  |  |  |  |
| Men | 208/1321 | 2973/16331 | 1.04 (0.90, 1.21) | 9 | 101/2664 | 1335/28807 | 0.87 (0.71, 1.08) | 8 |
| Women | 278/2122 | 2104/16745 | 1.04 (0.82, 1.33) | 8 | 173/4808 | 975/29963 | 1.14 (0.84, 1.55) | 12 |
| *P for interaction* |  |  | p = 0.16 |  |  |  | p = 0.56 |  |
| *Age* ^‡^ |  |  |  |  |  |  |  |  |
| < 65 years | 275/2499 | 2901/24170 | 1.00 (0.88, 1.13) | 7 | 49/4289 | 707/42921 | 0.83 (0.62, 1.12) | 8 |
| ≥ 65 years | 211/944 | 2176/8904 | 0.95 (0.72, 1.26) | 9 | 141/1424 | 1455/13125 | 1.06 (0.89, 1.26) | 10 |
| *P for interaction* |  |  | p = 0.51 |  |  |  | p = 0.11 |  |
| Excluding participants |  |  |  |  |  |  |  |  |
| [Excluding thyroid medication users at baseline](javascript:;) ^b^ | 411/2700 | 4715/27793 | 1.03 (0.87, 1.24) | 9 | 220/5835 | 2055/52199 | 1.01 (0.84, 1.21) | 11 |
| [Excluding thyroid medication users at baseline and during follow-up](javascript:;) ^b^ | 320/1730 | 3637/17778 | 1.10 (0.91, 1.33) | 6 | 123/2255 | 1342/18886 | 0.94 (0.64, 1.36) | 6 |
|  | **Stroke events ^a^** | | | | Stroke Mortality ^a^ | | | |
| *Model 1* | 258/ 3030 | 2394/29605 | 1.06 (0.92, 1.23) | 8 | 65 /5086 | 579/49321 | 1.15 (0.89, 1.50) | 10 |
| *Sex*^†^ |  |  |  |  |  |  |  |  |
| Men | 99/1199 | 1185/14983 | 1.09 (0.88, 1.34) | 8 | 30/1879 | 280/24455 | 1.40 (0.95, 2.06) | 5 |
| Women | 159/1831 | 1209/14922 | 1.03 (0.87, 1.22) | 8 | 35/3207 | 299/24867 | 1.10 (0.70, 1.71) | 10 |
| *P for interaction* |  |  | p = 0.34 |  |  |  | p = 0.15 |  |
| *Age* ^‡^ |  |  |  |  |  |  |  |  |
| < 65 years | 105/2061 | 1076/20259 | 1.02 (0.83, 1.25) | 5 | 18 /3714 | 194/36845 | 1.10 (0.66, 1.82) | 3 |
| ≥ 65 years | 153/969 | 1318/9344 | 1.12 (0.95, 1.33) | 8 | 47/1372 | 385/12475 | 1.25 (0.90, 1.73) | 10 |
| *P for interaction* |  | 0.16 | p = 0.15 |  |  |  | p = 0.91 |  |
| Excluding participants |  |  |  |  |  |  |  |  |
| [Excluding thyroid medication users at baseline](javascript:;) ^b^ | 217/2305 | 2145/24286 | 1.10 (0.96, 1.27) | 8 | 48/3934 | 498/43301 | 1.10 (0.81, 1.49) | 10 |
| [Excluding thyroid medication users at baseline and during follow-up](javascript:;) ^b^ | 159/1398 | 1562/14678 | 1.13 (0.90, 1.40) | 4 | 30/1401 | 292/14697 | 1.15 (0.64, 2.07) | 4 |

Abbreviations: CI, confidence interval; CHD, coronary heart disease; TPOAb, anti-thyroid peroxidase antibodies; HR, hazard ratio. A HR>1 indicates a higher hazard in the positive TPOAb group.

HRs were age, sex) adjusted. ^c^ Included cohorts with data on thyroid meds at follow up Aric, Cardiovascular Health Study, Leiden 85-plus, Tehran Thyroid, Rotterdam

^†^ Age adjusted ^‡^ These HRs were adjusted sex and age as a continuous variable.

In some Strata specific studies when they had an empty comparison group (no event in a strata).

^a^ Hunt excluded from the analysis because it measured TPOAb, with an indication for abnormal TSH.

^b^ In some strata specific studies were excluded when they had no events.

**eTable 3. Sensitivity Analyses: Association Between TPOAb Status with CHD Outcomes in Subclinical Hypothyroidism**

|  | Euthyroidism | SH With positive TPOAb Status | SH With Negative TPOAb Status | SH With Positive TPOAb vs Euthyroidism HR (95% CI) | SH With Negative TPOAb vs Euthyroidism  HR (95% CI) | SH With Positive TPOAb vs SH With Negative TPOAb  HR (95% CI*)* | p-value for HR positive vs. negative and no. of studies |
| --- | --- | --- | --- | --- | --- | --- | --- |
|  | **Events/**  **Participants** | **Events/**  **Participants** | **Events/**  **Participants** |  |  |  |  |
| *CHD events* |  |  |  |  |  |  |  |
| Random-effects model | 7341/65191 | 210/1627 | 297/1853 | 1.02 (0.85, 1.22) | 1.13 (0.97, 1.32) | 0.87 (0.72, 1.05) | p = 0.14; n = 10 |
| Excluding participants |  |  |  |  |  |  |  |
| [Excluding thyroid medication users at baseline](javascript:;) ^a^ | 6888/59027 | 173/1368 | 266/1586 | 0.96 (0.81, 1.14) | 1.12 (0.98, 1.29) | 0.85 (0.69, 1.04) | p = 0.12; n = 9 |
| [Excluding thyroid medication users at baseline or during follow-up](javascript:;) ^b^ | 4308/21065 | 72/495 | 143/750 | 0.99 (0.55, 1.45) | 1.02 (0.85, 1.22) | 0.87 (0.54, 1.40) | p = 0.11; n = 5 |
| Excluding participants with history of CVD ^§^ | 6304/ 60850 | 179/1482 | 261/1647 | 0.98 (0.83, 1.16) | 1.14 (0.99, 1.32) | 0.84 (0.69, 1.03) | p = 0.09; n = 8 |
| Excluding studies |  |  |  |  |  |  |  |
| [E](javascript:;)[xcluding study with recent iodine supplementation (SHIP)](javascript:;) | 7029/62242 | 209/1604 | 295/1812 | 1.02 (0.86, 1.22) | 1.13 (0.99, 1.29) | 0.85 (0.63, 1.08) | p = 0.16; n = 9 |
| Further adjustments in MV models ^c^ |  |  |  |  |  |  |  |
| Adjusted for age, sex, systolic blood pressure, smoking status, total cholesterol, and diabetes at baseline (MV model 1) | 7082/62092 | 208/1592 | 290/1788 | 0.94 (0.76, 1.16) | 1.09 (0.93, 1.27) | 0.86 (0.71, 1.05) | p = 0.13; n = 9 |
| MV model 1 + lipid-lowering and antihypertensive medications | 5153/31510 | 163/1063 | 233/1315 | 0.93 (0.79, 1.08) | 1.02 (0.86, 1.22) | 0.91 (0.74, 1.12) | p = 0.36; n = 6 |
| MV model 1 + BMI | 7082/62092 | 208/1592 | 290/1788 | 0.93 (0.76, 1.13) | 1.09 (0.92, 1.30) | 0.87 (0.72, 1.07) | p = 0.19; n= 10 |
| *CHD mortality* |  |  |  |  |  |  |  |
| Random-effects model | 3122/93984 | 93/2188 | 173/3115 | 1.18 (0.81, 1.72) | 1.23 (1.03, 1.48) | 0.88 (0.64, 1.21) | p = 0.43; n = 9 |
| Excluding participants |  |  |  |  |  |  |  |
| [Excluding thyroid medication users at baseline](javascript:;) ^a^ | 2843/86353 | 70/1755 | 152/2705 | 1.13 (0.74, 1.74) | 1.22 (1.01, 1.49) | 0.78 (0.57, 1.07) | p = 0.12; n = 7 |
| [Excluding thyroid medication users at baseline or during follow-up](javascript:;) ^b^ | 1480/21412 | 22/503 | 73/784 | 0.81 (0.52, 1.26) | 1.32 (0.75, 2.33) | 0.60 (0.35, 1.01) | p = 0.06; n=3 |
| Excluding participants with history of CVD § | 2266/86165 | 65/1979 | 113/2746 | 1.08 (0.73, 1.60) | 1.18 (0.95, 1.48) | 0.82 (0.59, 1.15) | p = 0.25; n=8 |
| Excluding studies |  |  |  |  |  |  |  |
| [Excluding study with recent iodine supplementation (SHIP)](javascript:;) | 3019/90377 | 93/2158 | 173/3068 | 1.20 (0.80, 1.79) | 1.23 (1.03, 1.48) | 0.90 (0.64, 1.27) | p = 0.56; n= 8 |
| Further adjustments in MV models ^c^ |  |  |  |  |  |  |  |
| Adjusted for age, sex, systolic blood pressure, smoking status, total cholesterol, and diabetes at baseline (MV model 1) | 2995/89596 | 92/2137 | 167/3029 | 1.22 (0.77, 1.94) | 1.17 (0.97, 1.41) | 0.91 (0.63, 1.32) | p = 0.62; n= 8 |
| MV model 1 + lipid-lowering and antihypertensive medications | 2075/48515 | 73/1489 | 130/2262 | 1.24 (0.77, 2.84) | 1.19 (0.89, 1.60) | 1.01 (0.65, 1.55) | p = 0.99; n= 5 |
| MV model 1 + BMI | 2995/89596 | 92/2137 | 167/3029 | 1.16 (0.76, 1.79) | 1.17 (0.97, 1.41) | 0.83 (0.62, 1.12) | p = 0.22; n= 8 |

Abbreviations: MV, multivariate; NA, not applicable; SH, subclinical hypothyroidism; BMI, Body mass index; CVD, cardiovascular disease; HR, hazard ratio.

HR were adjusted for age, sex and TSH (log scale). A HR>1 indicates a higher hazard in the positive TPOAb group.

^a^ The numbers of thyroid medication users (T4, antithyroid drugs) at baseline and during follow-up are reported in Table 1.

^b^ Some participants were excluded from the MV models because of lack of data on covariates.

^C^ Included cohorts with data on thyroid meds at follow up Aric, Cardiovascular Health Study, Leiden 85-plus, Tehran Thyroid, Rotterdam

^§^Cardiovascular disease at baseline was defined as a known history of stroke, transient ischemic attack, myocardial infarction, angina pectoris, coronary angioplasty, or bypass surgery.

**eTable 4. Sensitivity Analyses: Association Between TPOAb Status with Stroke Outcomes in Subclinical Hypothyroidism**

|  | Euthyroidism | SH With positive TPOAb Status | SH With Negative TPOAb Status | SH With Positive TPOAb vs Euthyroidism HR (95% CI) | SH With Negative TPOAb vs Euthyroidism HR (95% CI) | SH With Positive TPOAb vs SH With Negative TPOAb  HR (95% CI) | p-value for HR positive vs. negative and no. of studies |
| --- | --- | --- | --- | --- | --- | --- | --- |
|  | **Events/**  **Participants** | **Events/**  **Participants** | **Events/**  **Participants** |  |  |  |  |
| *Stroke Events* | | | | | | | |
| Random-effects model | 2498/32910 | 72/967 | 124/1133 | 0.94 (0.70, 1.23) | 1.24 (0.99, 1.55) | 0.68 (0.51, 0.90) | p = 0.01; n = 4 |
| Excluding participants |  |  |  |  |  |  |  |
| [Excluding thyroid medication users at baseline](javascript:;) ^a^ | 2640/29935 | 60/790 | 134/1052 | 0.87 (0.62, 1.22) | 1.29 (1.01, 1.63) | 0.63 (0.45, 0.87) | p = 0.06; n= 4 |
| [Excluding thyroid medication users at baseline or during follow-up](javascript:;) ^b^ | 1875/17628 | 32/388 | 87/635 | 0.75 (0.58, 1.24) | 1.29 (1.08, 1.91) | 0.63 (0.40, 0.97) | p = 0.04; n=3 |
| Excluding participants with history of CVD | 2429/32163 | 71/942 | 119/1104 | 1.01 (0.66, 1.26) | 1.14 (0.89, 1.47) | 0.67 (0.49, 0.92) | p = 0.01; n= 5 |
| Excluding studies |  |  |  |  |  |  |  |
| [Excluding study with recent iodine supplementation (SHIP)](javascript:;) | 2717/33102 | 83/1028 | 151/1268 | 0.94 (0.70, 1.27) | 1.24 (0.99, 1.55) | 0.68 (0.51, 0.90) | p = 0.01; n= 5 |
| Further adjustments in MV models ^c^ |  |  |  |  |  |  |  |
| Adjusted for age, sex, systolic blood pressure, smoking status, total cholesterol, and diabetes at baseline (MV model 1) | 2816/33148 | 81/1008 | 147/1253 | 0.87 (0.64, 1.17) | 1.22 (0.97, 1.54) | 0.65 (0.48, 0.87) | p = 0.01; n= 5 |
| MV model 1 + lipid-lowering and antihypertensive medications | 2388/ 27685 | 76/ 917 | 139/1172 | 0.87 (0.64, 1.19) | 1.22 (0.96, 1.55) | 0.63 (0.46, 0.85) | p = 0.01; n= 3 |
| MV model 1 + BMI | 2775/32841 | 79/998 | 146/1243 | 0.87 (0.64, 1.19) | 1.23 (0.98, 1.55) | 0.64 (0.47, 0.86) | p = 0.01; n= 5 |
| Stroke mortality | | | | | | | |
| Random-effects model | 757/61465 | 18/1516 | 30/2362 | 1.14 (0.67, 1.93) | 1.33 (0.87, 2.04) | 0.73 (0.38, 1.39) | p = 0.28; n= 6 |
| Excluding participants |  |  |  |  |  |  |  |
| [Excluding thyroid medication users at baseline](javascript:;) ^a^ | 654/54024 | 14/1147 | 26/1973 | 1.30 (0.71, 2.39) | 1.50 (0.94, 2.37) | 0.69 (0.32, 1.49) | p = 0.34; n=6 |
| [Excluding thyroid medication users at baseline or during follow-up](javascript:;) ^b^ | 367/17649 | 5/388 | 13/640 | 1.58 (0.59, 4.24) | 1.72 (0.92, 3.21) | 0.85 (0.17, 4.21) | p = 0.83; n=3 |
| Excluding participants with history of CVD | 602/54768 | 16/1366 | 22/2046 | 1.26 (0.72, 2.21) | 1.31 (0.80, 2.14) | 0.69 (0.34, 1.42) | p = 0.31; n=5 |
| Excluding studies |  |  |  |  |  |  |  |
| [Excluding study with recent iodine supplementation (SHIP)](javascript:;) | 702/57715 | 17/1484 | 30/2314 | 1.10 (0.64, 1.90) | 1.33 (0.87, 2.04) | 0.69 (0.36, 1.36) | p = 0.28; n=6 |
| Further adjustments in MV models ^c^ |  |  |  |  |  |  |  |
| Adjusted for age, sex, systolic blood pressure, smoking status, total cholesterol, and diabetes at baseline (MV model 1) | 721/57274 | 18/1467 | 29/2279 | 1.15 (0.67, 1.96) | 1.29 (0.84, 1.99) | 0.64 (0.32, 1.31) | p = 0.22; n=4 |
| MV model 1 + lipid-lowering and antihypertensive medications | 583/41246 | 15/1246 | 27/1939 | 1.04 (0.58, 1.85) | 1.29 (0.82, 2.02) | 0.74 (0.30, 1.83) | p = 0.51; n=6 |
| MV model 1 + BMI | 721/57274 | 18/1467 | 29/2279 | 1.21 (0.71, 2.07) | 1.34 (0.86, 2.06) | 0.66 (0.33, 1.36) | p = 0.26; n=4 |

Abbreviations: MV, multivariate; NA, not applicable; SH, subclinical hypothyroidism; BMI, Body mass index; CVD, cardiovascular disease; HR, hazard ratio.

HR were adjusted for age, sex and TSH (log scale). A HR>1 indicates a higher hazard in the positive TPOAb group.

^a^ The numbers of thyroid medication users (T4, antithyroid drugs) at baseline and during follow-up are reported in Table 1.

^b^ Some participants were excluded from the MV models because of lack of data on covariates.

^C^ Included cohorts with data on thyroid meds at follow up Aric, Cardiovascular Health Study, Leiden 85-plus, Tehran Thyroid, Rotterdam

^§^ Cardiovascular disease at baseline was defined as a known history of stroke, transient ischemic attack, myocardial infarction, angina pectoris, coronary angioplasty, or bypass surgery.

**eTable 5. Quality Assessment of Included Cohorts Using the Newcastle-Ottawa Scale**

| **Study** | **Selection** | | | | **Comparability** | **Outcome** | | |  |
| --- | --- | --- | --- | --- | --- | --- | --- | --- | --- |
|  | **Representativeness of exposed cohort** | **Selection of the non-exposed cohort** | **Ascertainment of exposure** | **Demonstration of the outcome of interest was not present at start of the study†** | **Comparability of cohorts on the basis of the design or analysis‡** | **Assessment of outcome** | **Was follow up long enough for outcomes to occur** | **Adequacy of follow up (Missing data on primary outcome at follow-up)** | **Quality Score** |
| Aric Study | * | * | * | * | ** | * | * | * | 9 |
| Bari Study | - | * | * | - | ** | * | * | * | 7 |
| Di@bet.es Study 2009 | * | * | * | * | ** | * | * | * | 9 |
| Busselton Health Study | * | * | * | * | ** | * | * | * | 9 |
| Cardiovascular Health Study | * | * | * | * | ** | * | * | * | 9 |
| ELSA-Brasil Study | * | * | * | * | ** | * | * | * | 9 |
| Japanese Brazilian Thyroid Study | * | * | * | * | ** | * | * | * | 9 |
| HUNT Study 1995 | * | * | * | - | ** | * | * | * | 8 |
| Leiden 85-plus Study | * | * | * | * | * | * | * | * | 8 |
| NHANES (1999-2002, 2007-2012) | * | * | * | * | ** | * | * | * | 9 |
| PREVEND Study | * | * | * | * | ** | * | * | * | 9 |
| Rotterdam Study | * | * | * | * | ** | * | * | * | 9 |
| SHIP Study | * | * | * | * | ** | - | * | * | 8 |
| Tehran Thyroid Study | * | * | * | * | ** | * | * | * | 9 |

**eFigure 6. The proportional hazards assumption of the Cox model assessed at the study level with graphical methods based on Schoenfeld residuals.**


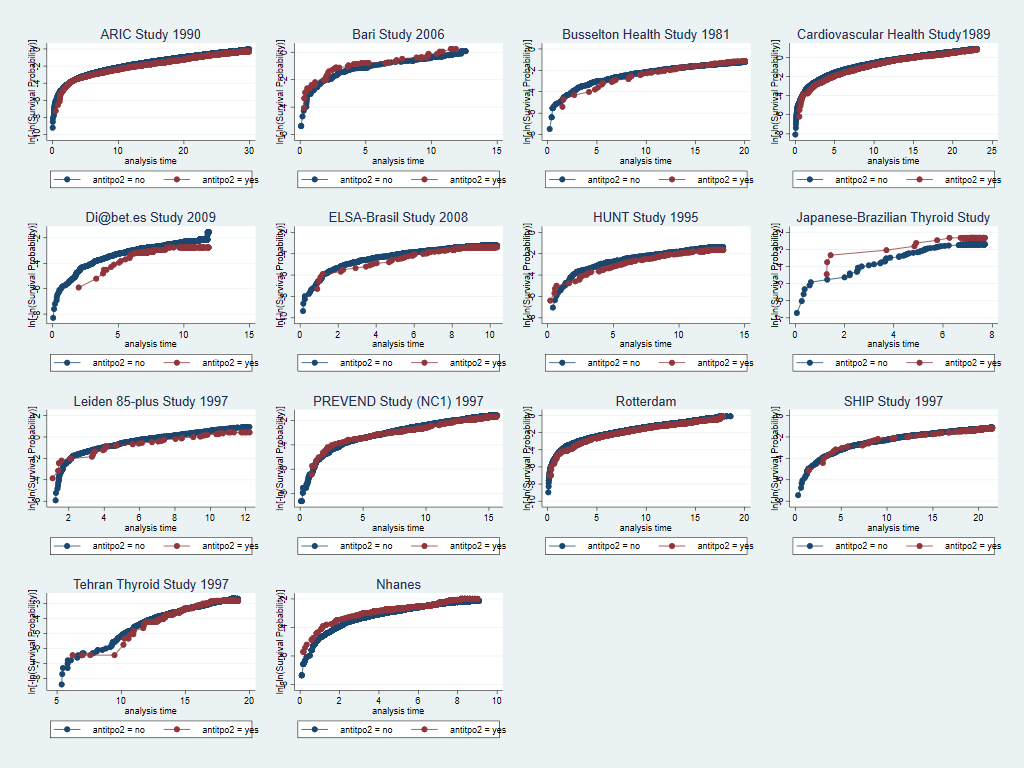


| Study | Variable | Rho | Chi2 | Prob>Chi2 |
| --- | --- | --- | --- | --- |
| ARIC Study 1990 | global | 0.01509 | 1.34 | 0.2462 |
| Bari Study 2006 | global | 0.02105 | 0.05 | 0.8181 |
| Busselton Health Study 1981 | global | 0.0719 | 2.66 | 0.1028 |
| Cardiovascular Health Study 1989 | global | 0.0295 | 2.87 | 0.0901 |
| Di@bet.es Study 2009 | global | 0.04176 | 0.53 | 0.4679 |
| ELSA-Brasil Study 2008 | global | 0.07118 | 2.34 | 0.126 |
| HUNT Study 1995 | global | 0.03656 | 0.45 | 0.5035 |
| Japanese Brazilian Thyroid Study | global | 0.04043 | 0.1 | 0.7523 |
| Leiden 85-plus Study 1997 | global | 0.03705 | 0.51 | 0.475 |
| PREVEND Study (NC1) 1997 | global | -0.01 | 0.09 | 0.758 |
| Rotterdam | global | 0.00147 | 0.01 | 0.9392 |
| SHIP Study 1997 | global | 4e-05 | 0 | 0.9994 |
| Tehran Thyroid Study 1997 | global | 0.00091 | 0 | 0.9882 |
| Nhanes | global | 0.02579 | 0.5 | 0.4789 |

**eFigure 7. PRISMA (Preferred Reporting Items for Systematic Reviews and Meta-Analyses) Checklist**

| **Section and Topic** | **Item #** | **Checklist** | **Location where item is reported** |
| --- | --- | --- | --- |
| **TITLE** | | |  |
| Title | 1 | Identify the report as a systematic review. | Page 7 |
| **ABSTRACT** | | |  |
| Abstract | 2 | See the PRISMA 2020 for Abstracts checklist. | Page 4 |
| **INTRODUCTION** | | |  |
| Rationale | 3 | Describe the rationale for the review in the context of existing knowledge. | Page 4, 7 |
| Objectives | 4 | Provide an explicit statement of the objective(s) or question(s) the review addresses. | Page 4,7 |
| **METHODS** | | |  |
| Eligibility criteria | 5 | Specify the inclusion and exclusion criteria for the review and how studies were grouped for the syntheses. | Page 7 |
| Information sources | 6 | Specify all databases, registers, websites, organisations, reference lists and other sources searched or consulted to identify studies. Specify the date when each source was last searched or consulted. | Page 7 |
| Search strategy | 7 | Present the full search strategies for all databases, registers and websites, including any filters and limits used. | Page 7, Supplementary page 3-4 |
| Selection process | 8 | Specify the methods used to decide whether a study met the inclusion criteria of the review, including how many reviewers screened each record and each report retrieved, whether they worked independently, and if applicable, details of automation tools used in the process. | Page 8 |
| Data collection process | 9 | Specify the methods used to collect data from reports, including how many reviewers collected data from each report, whether they worked independently, any processes for obtaining or confirming data from study investigators, and if applicable, details of automation tools used in the process. | Page 8 |
| Data items | 10a | List and define all outcomes for which data were sought. Specify whether all results that were compatible with each outcome domain in each study were sought (e.g. for all measures, time points, analyses), and if not, the methods used to decide which results to collect. | Page 7-9, Supplementary page 4 |
|  | 10b | List and define all other variables for which data were sought (e.g. participant and intervention characteristics, funding sources). Describe any assumptions made about any missing or unclear information. | Page 8 |
| Study risk of bias assessment | 11 | Specify the methods used to assess risk of bias in the included studies, including details of the tool(s) used, how many reviewers assessed each study and whether they worked independently, and if applicable, details of automation tools used in the process. | Page 11 |
| Effect measures | 12 | Specify for each outcome the effect measure(s) (e.g. risk ratio, mean difference) used in the synthesis or presentation of results. | Page 10 |
| Synthesis methods | 13a | Describe the processes used to decide which studies were eligible for each synthesis (e.g. tabulating the study intervention characteristics and comparing against the planned groups for each synthesis (item #5)). |  |
|  | 13b | Describe any methods required to prepare the data for presentation or synthesis, such as handling of missing summary statistics, or data conversions. | Page 11 |
|  | 13c | Describe any methods used to tabulate or visually display results of individual studies and syntheses. | Page 9-11 |
|  | 13d | Describe any methods used to synthesize results and provide a rationale for the choice(s). If meta-analysis was performed, describe the model(s), method(s) to identify the presence and extent of statistical heterogeneity, and software package(s) used. | Page 9-11 |
|  | 13e | Describe any methods used to explore possible causes of heterogeneity among study results (e.g. subgroup analysis, meta-regression). | Page 12 |
|  | 13f | Describe any sensitivity analyses conducted to assess robustness of the synthesized results. | Page 12 |
| Reporting bias assessment | 14 | Describe any methods used to assess risk of bias due to missing results in a synthesis (arising from reporting biases). | Page 11 |
| Certainty assessment | 15 | Describe any methods used to assess certainty (or confidence) in the body of evidence for an outcome. | Page 11 |
| **RESULTS** | | |  |
| Study selection | 16a | Describe the results of the search and selection process, from the number of records identified in the search to the number of studies included in the review, ideally using a flow diagram. | Page 12 and Supplementary page 4 |
|  | 16b | Cite studies that might appear to meet the inclusion criteria, but which were excluded, and explain why they were excluded. | Supplementary page 4 |
| Study characteristics | 17 | Cite each included study and present its characteristics. | Table 1 of results |
| Risk of bias in studies | 18 | Present assessments of risk of bias for each included study. | Supplementary eTable 5 |
| Results of individual studies | 19 | For all outcomes, present, for each study: (a) summary statistics for each group (where appropriate) and (b) an effect estimate and its precision (e.g. confidence/credible interval), ideally using structured tables or plots. | Page 12 and 13 |
| Results of syntheses | 20a | For each synthesis, briefly summarise the characteristics and risk of bias among contributing studies. | Page 12 and 13 |
|  | 20b | Present results of all statistical syntheses conducted. If meta-analysis was done, present for each the summary estimate and its precision (e.g. confidence/credible interval) and measures of statistical heterogeneity. If comparing groups, describe the direction of the effect. | Page 12 and 13 |
|  | 20c | Present results of all investigations of possible causes of heterogeneity among study results. | Page 12 and 13 |
|  | 20d | Present results of all sensitivity analyses conducted to assess the robustness of the synthesized results. | Page 12 and 13 |
| Reporting biases | 21 | Present assessments of risk of bias due to missing results (arising from reporting biases) for each synthesis assessed. | page 13,15 |
| Certainty of evidence | 22 | Present assessments of certainty (or confidence) in the body of evidence for each outcome assessed. | Page 15 |
| **DISCUSSION** | | |  |
| Discussion | 23a | Provide a general interpretation of the results in the context of other evidence. | Page 15 |
|  | 23b | Discuss any limitations of the evidence included in the review. | Page 15 |
|  | 23c | Discuss any limitations of the review processes used. | Page 16 |
|  | 23d | Discuss implications of the results for practice, policy, and future research. | Page 16 |
| **OTHER INFORMATION** | | |  |
| Registration and protocol | 24a | Provide registration information for the review, including register name and registration number, or state that the review was not registered. | Page 7 |
|  | 24b | Indicate where the review protocol can be accessed, or state that a protocol was not prepared. | Page 7 |
|  | 24c | Describe and explain any amendments to information provided at registration or in the protocol. | NA |
| Support | 25 | Describe sources of financial or non-financial support for the review, and the role of the funders or sponsors in the review. | Page 18 |
| Competing interests | 26 | Declare any competing interests of review authors. | Page 18 |
| Availability of data, code and other materials | 27 | Report which of the following are publicly available and where they can be found: template data collection forms; data extracted from included studies; data used for all analyses; analytic code; any other materials used in the review. | Page 18 |
